# Hype um die Vitamin-D-Substitution: Was bleibt?

**DOI:** 10.1007/s00108-020-00869-y

**Published:** 2020-09-24

**Authors:** Heike A. Bischoff-Ferrari

**Affiliations:** Klinik für Geriatrie, Universitätsspital Zürich, Universitäre Klinik für Akutgeriatrie, Stadtspital Waid &Triemli, Universität Zürich, RAE B, Rämistr. 100, 8091 Zürich, Schweiz

**Keywords:** Vitamin-D-Mangel, Nahrungsergänzungen, Knochenfrakturen, Krebs, Kardiovaskuläre Erkrankungen, Vitamin D deficiency, Food supplementations, Fractures, bone, Cancer, Cardiovascular diseases

## Abstract

Der „Hype um Vitamin D“ ist neben der Bedeutung für die Knochengesundheit auch auf die ubiquitäre Präsenz des Vitamin-D-Rezeptors in vielen Organsystemen zurückzuführen. Große Beobachtungsstudien lieferten Hinweise, dass ein Vitamin-D-Mangel Risiken altersassoziierte chronische Erkrankungen, wie Krebs- und kardiovaskuläre Erkrankungen, begünstigt. In der vorliegenden Übersicht werden neueste Informationen, einerseits zur Knochengesundheit bei erwachsenen Menschen sowie andererseits zu Krebs- und kardiovaskulären Erkrankungen, anhand der aktuellen Resultate des großen Vitamin D and Omega‑3 Trial (VITAL) eingeordnet.

## Abstract (en)

The hype about vitamin D can be traced back to the ubiquitous presence of vitamin D receptors in many organ systems, in addition to the importance for healthy bones. Large observational studies have provided indications that a vitamin D deficiency favors risks for age-associated chronic diseases, such as cancer and cardiovascular diseases. In this article the latest information on bone health in adult persons as well as cancer and cardiovascular diseases is summarized based on the current results of the large vitamin D and omega‑3 trial (VITAL).

Die traditionelle Rolle von Vitamin D und die Grundlage der Einnahmeempfehlungen zu Vitamin D beziehen sich auf die Knochengesundheit. Der „Hype um Vitamin D“ bezieht sich darüber hinaus auf die ubiquitäre Präsenz des Vitamin-D-Rezeptors in vielen Organsystemen sowie Hinweise aus großen Beobachtungsstudien, dass ein Vitamin-D-Mangel Risiken für altersassoziierte chronische Erkrankungen, wie Krebs- und kardiovaskuläre Erkrankungen, begünstigt [1]. Diese Literatur, kombiniert mit Studien, die zeigten, dass jeder zweite Erwachsene [2] und jedes zweite Kind [3] einen Vitamin-D-Mangel haben, triggerten randomisierte placebokontrollierte Interventionsstudien zur Klärung eines kausalen Zusammenhangs. In der vorliegenden Übersicht werden diesbezüglich neue Informationen, einerseits zur Knochengesundheit bei erwachsenen Menschen sowie andererseits zu Krebs- und kardiovaskulären Erkrankungen anhand der neuesten Resultate des großen Vitamin D and Omega‑3 Trial (VITAL), eingeordnet.

## Aktuelle Empfehlungen zu Vitamin D bezogen auf die Knochengesundheit

In den aktuellen Empfehlungen zu Vitamin D (Institute of Medicine [4], Deutsche Gesellschaft für Ernährung (DGE, [5]), Bundesamt für Gesundheit Schweiz (BAG Schweiz, [6]), US Endocrine Society [7], International Osteoporosis Foundation (IOF, [8])) wird die tägliche Vitamin-D-Zufuhr altersabhängig definiert: 400 Internationale Einheiten (IE)/Tag im 1. Lebensjahr, 600 IE/Tag zwischen dem 2. und 64. Lebensjahr sowie 800 IE/Tag ab dem 65. Lebensjahr (in der Schweiz 800 IE/Tag ab dem 60. Lebensjahr).

In über 97 % der Fälle korrigiert die Gabe von 600–800 IE Vitamin D/Tag einen Mangel bei Erwachsenen

Es ist gut belegt, dass im Erwachsenenalter 600–800 IE/Tag in über 97 % der Fälle den Vitamin-D-Mangel korrigieren können [9, 10]. Diese Dosis ist zudem auf die Population bezogen sicher und ohne vorherige Messung der 25-Hydroxy-Vitamin-D-Blutkonzentration anwendbar (Institute of Medicine [4], DGE [5], BAG Schweiz [6], US Endocrine Society [7], IOF [8]).

## Vitamin D and Omega-3 Trial

Bezüglich kardiovaskulärer Wirkungsmechanismen zeigen mechanistische Studien, dass glatte Gefäßmuskelzellen, Endothelzellen und Kardiomyozyten den Vitamin-D-Rezeptor exprimieren [11–14], und dass Vitamin D das Renin-Angiotensin-Aldosteron-System über die Unterdrückung der Reningenexpression reguliert [15, 16]. Bezüglich der Karzinogenese gibt es Hinweise, dass Vitamin D das unkontrollierte Zellwachstum hemmt [1] und die Immunabwehr stärkt [1, 17, 18]. Konsistent mit einer Wirkung von Vitamin D über die Knochengesundheit hinaus, fand eine große sequenzielle Metaanalyse aller Interventionsstudien zu Vitamin D, verglichen mit Placebo, eine signifikante 4 %ige Reduktion der Gesamtmortalität [19].

Das VITAL [20, 21] untersuchte daher in einer großen doppelblind randomisierten Interventionsstudie 25.871 erwachsene Menschen, 12.786 Männer ab 50 Jahren und 13.085 Frauen ab 55 Jahren. Verglichen wurde 2000 IE Vitamin D/Tag mit Placebo über einen Zeitraum von durchschnittlich 5,3 Jahren. Alle Teilnehmer konnten zusätzlich zur Studienmedikation bis 800 IE Vitamin D einnehmen und wurden auf das Auftreten von Krebs und kardiovaskulären Ereignissen überwacht. Zu Studienbeginn hatten nur 12,7 % einen Vitamin-D-Mangel mit 25-Hydroxy-Vitamin-D-Blutwerten unter 20 ng/ml, und der Mittelwert betrug 31 ng/ml. Während der medianen Nachbeobachtungszeit von 5,3 Jahren fanden die Forscher über alle Teilnehmer hinweg keine signifikanten Unterschiede zwischen der Vitamin‑D- und der Placebogruppe in Bezug auf die Inzidenz schwerwiegender kardiovaskulärer-Ereignisse (d. h. Myokardinfarkt, Schlaganfall oder kardiovaskulär bedingter Tod) oder Krebserkrankungen. Die Gesamtmortalitäten waren in beiden Gruppen identisch (3,8 %).

### Krebserkrankungen

Bezüglich Krebserkrankungen zeigte sich eine kleine nichtsignifikante Reduktion bei der Vitamin-D-Gruppe (793 Krebserkrankungen bei den 12.927 Teilnehmern unter Vitamin D, verglichen mit 824 Krebserkrankungen bei den 12.944 Teilnehmern unter Placebo). Zusätzlich war die Krebsmortalität in der Vitamin-D-Gruppe signifikant, um 17–25 %, vermindert. Die größte Verringerung der Krebstodesfälle mit 25 % zeigte sich nach Ausschluss der ersten beiden Jahre als gängige Praxis bei der Analyse von Daten aus Studien mit Nahrungsergänzungsmitteln und Krebs, da erwartet wird, dass sich die Auswirkungen von Ernährungsfaktoren auf das Krebsrisiko, langsam entwickeln und erst nach mehreren Jahren deutlich werden.

Die Gesamtmortalitäten waren in der Vitamin-D- und der Placebogruppe identisch

Außerdem zeigte sich unter Vitamin-D-Gabe, verglichen zu Placebo, eine signifikante Reduktion des Krebsrisiko um 23 % bei der Subgruppe der afroamerikanischen Teilnehmer, die anhand populationsbasierter Erhebungen in den USA das größte Risiko einer Vitamin-D-Unterversorgung haben.

#### Herz-Kreislauf-Erkrankung

Bezüglich aller kardiovaskulären Ereignissen zeigte sich ebenfalls eine kleine, nichtsignifikante Reduktion unter Vitamin D (bei den Teilnehmern der Vitamin-D-Gruppe traten insgesamt 396 kardiovaskuläre Ereignisse auf, verglichen mit 409 in der Placebogruppe). Weder das Auftreten von Herzinfarkt, Schlaganfall oder kardiovaskulärem Tod, einzeln betrachtet, noch die Mortalität waren in der Vitamin-D-Gruppe gegenüber Placebo vermindert.

#### Zusammenfassung

Die große Interventionsstudie VITAL in den USA zeigte keinen Benefit bezüglich der Prävention von Herz-Kreislauf-Erkrankungen unter einer langjährigen Supplementation mit Vitamin D, verglichen zu Placebo. Allerdings kann anhand von VITAL eine Risikoreduktion bezüglich Krebserkrankungen bei Afroamerikanern um 23 % und Krebsmortalität um bis zu 25 %, unabhängig von der Ethnizität, unter einer zusätzlichen Einnahme von 2000 IE Vitamin D am Tag, nicht ausgeschlossen werden. Bei der Einordung der VITAL-Resultate ist wichtig, dass über 85 % der Studienteilnehmer zu Beginn keinen Vitamin-D-Mangel hatten und zudem alle Studienteilnehmer (auch die Placebogruppe) zusätzlich 800 IE Vitamin D/Tag einnehmen durften. Damit besteht ein sehr konservatives Studiendesign, was einen Benefit von Vitamin D auf die Endpunkte Krebs und kardiovaskuläre Erkrankungen, insbesondere bei Menschen mit Vitamin-D-Mangel, nicht ausschließen kann. Möglicherweise war auch die Beobachtungszeit für diese schwerwiegenden Ereignisse nicht ausreichend, um die Wirkung eines Nahrungsergänzungsmittels zu erfassen. Das VITAL wird weitere Daten zu den sekundären Endpunkten liefern, die mit Spannung erwartet werden dürfen.

## Frakturprävention bei Erwachsenen 50+

Die neuesten Metaanalysen klinischer Interventionsstudien zu Vitamin-D-Supplementation und Frakturreduktion hinterfragen die bestehenden Richtlinien zu Vitamin D bei Erwachsenen. Grundsätzlich begünstigt ein Vitamin-D-Mangel belegtermaßen Stürze [22], Knochenabbau und Frakturen [23], und das besonders bei Erwachsenen im Alter von 65 Jahren und älter. In den Jahren 2016–2018 wurden 4 Metaanalysen durchgeführt, um den Nutzen von Vitamin D für die Frakturprävention zu untersuchen. Zwei dieser Metaanalysen konzentrieren sich auf die Primärprävention von Frakturen bei Erwachsenen ab 50 Jahren, bei denen kein Risiko für Frakturen und kein Vitamin-D-Mangel besteht [24, 25]. Eine weitere Metaanalyse konzentrierte sich auf die Kombination von Vitamin D plus Kalzium [26] und die zuletzt publizierte Metaanalyse auf die individuelle Wirkung von Vitamin D ohne Kalzium [27].

### Metaanalyse Frakturprävention bei Menschen im Alter 65+ bezüglich Vitamin D plus Kalzium [26]

Die 2016 von Weaver et al. veröffentlichte Metaanalyse [26] hatte zum Ziel, die kombinierte Wirkung von Vitamin D mit Kalzium im Vergleich zu Placebo zu untersuchen. Bei dieser Auswahl von klinischen Studien umfasste diese Metaanalyse etwa 40 % der qualitativ hochwertigen Daten zur Frakturreduktion mithilfe von Vitamin D, die zu den aktuellen Leitlinien zu Vitamin D beitrugen. Insgesamt fassten die Autoren 8 randomisierte kontrollierte Studien zusammen (*n* = 30.970 Erwachsene, überwiegendes Alter 65+) und fanden eine signifikante 15 %ige Reduktion aller Frakturen (RR = 0,85; 95 %-Konfidenzintervall [95 %-KI] 0,73–0,98) und eine signifikante 30 %ige Reduktion von Hüftfrakturen (RR = 0,70, 95 %-KI 0,56–0,87; [26]). Die Kombination mit Kalzium führte dazu, dass Studien mit Bolusgaben von Vitamin D ausgeschlossen wurden, und die meisten der eingeschlossenen Studien die heute empfohlene Dosis von 800 IE Vitamin D/Tag untersuchten. Außerdem war der Großteil der Studienpopulation im Alter von 65 Jahren und älter und hatte damit ein erhöhtes Risiko für Stürze, Frakturen und Vitamin-D-Mangel. Die Autoren schlossen zudem sowohl zu Hause lebende als auch institutionalisierte ältere Erwachsene ein, was den Anteil der Hochrisikopopulation für Vitamin-D-Mangel erhöhte.

### Frakturprävention bei zu Hause lebenden Erwachsenen ab 50 Jahren ohne Osteoporose [24] bezüglich Vitamin D mit oder ohne Kalzium

Die 2017 von Zhao et al. veröffentlichte Metaanalyse [24] hatte zum Ziel, die Wirkung von Vitamin D und Kalzium individuell sowie deren Kombination auf die Primärprävention von Frakturen bei zu Hause lebenden Erwachsenen im Alter 50+ Jahren zu bewerten. Die Autoren schlossen 33 Studien mit insgesamt 51.145 Männern und Frauen ein [24]. Der primäre Endpunkt war Hüftfrakturen, und zu den sekundären Endpunkten gehörten jegliche nichtvertebrale Frakturen, vertebrale Frakturen und alle Frakturen. In Bezug auf die Studienqualität schlossen die Autoren jede randomisierte klinische Studie mit einer Placebo-Kontroll-Gruppe oder ohne Behandlung in der Kontrollgruppe ein.

Die Autoren fanden keine signifikante Risikoreduktion von Vitamin D oder Kalzium bezogen auf das Hüftbruchrisiko im Vergleich zu Placebo oder keiner Kontrollintervention (Vitamin D: „risk ratio“ [RR]: 1,21, 95 %-KI 0,99–1,47; Kalzium: RR: 1,53, 95 %-KI 0,97–2,42). Auch zeigte sich keine signifikante Risikoreduktion durch die kombinierte Gabe von Vitamin D plus Kalzium bezüglich des Hüftbruchrisikos (RR: 1,09, 95 %-KI 0,85–1,39). Dazu fanden die Autoren keinen Benefit für andere Frakturendpunkte, weder für die individuellen Supplemente noch deren Kombination. Die Autoren führten zudem mehrere Untergruppenanalysen durch und dokumentierten, dass ihre Ergebnisse i. Allg. konsistent waren, unabhängig von Vitamin-D- oder Kalziumdosis, Geschlecht, vorbestehender Fraktur, Zufuhr von Kalzium aus der Ernährung und 25-Hydroxy-Vitamin-D-Blutspielgel. Die Autoren folgerten, dass die Routineanwendung von Kalzium, Vitamin D und deren Kombination bei zu Hause lebenden Menschen im Alter 50+ und bezogen auf die Primärprävention von Knochenbrüchen von ihren Resultaten nicht unterstützt wird.

Die Methodik und Konklusionen der Autoren wurden bezüglich verschiedener Aspekte kritisiert [28]:

- *Erstens* wählten die Autoren unter Einbeziehung von Studien bei Erwachsenen ab 50 Jahren und Ausschluss älterer Erwachsener in Institutionen eine Zielgruppe aus, die weniger anfällig für einen Vitamin-D-Mangel war und ein geringes Frakturrisiko hatte.
- *Zweitens* schlossen die Autoren viele Studien ein, die wenig Gelegenheit hatten, einen Nutzen der Interventionen bezüglich Frakturen zu demonstrieren. In einem Drittel der Studien war das Follow-up zu kurz (11 von 33 mit Follow-ups ≤12 Monate). Außerdem wurden 4 Studien mit einem qualitativ minderwertigen Studiendesign ohne Verblindung in der Kontrollgruppe eingeschlossen.
- *Drittens* wurde die Adhärenz bezüglich Studienmedikation nicht berücksichtigt, obgleich der Einfluss der Adhärenz in früheren Metaanalysen als signifikant belegt wurde [23, 29]. Bemerkenswerterweise nahmen in der stark gewichteten RECORD(„Randomised Evaluation of Calcium Or vitamin D“)-Studie nur etwa die Hälfte der Teilnehmer ihre Vitamin D- oder Kalziumpräparate ein [30].
- *Viertens* gaben 8 der 12 eingeschlossenen Studien zu Vitamin D das Supplement in Bolusdosen (oral oder i.m.), was in der Literatur wiederholt Bedenken hinsichtlich der Förderung von Stürzen und Frakturen aufwirft [31, 32]. Insbesondere wurde verpasst, die Bolusstudien in einer Subgruppenanalyse separat auszuwerten.

### Frakturprävention bei Erwachsenen im Alter von 50+ Jahren ohne Osteoporose und ohne Vitamin-D-Mangel bezüglich Vitamin D mit und ohne Kalzium

Die US-Arbeitsgruppe für Präventivmaßnahmen (US Preventive Task Force) hat 2018 sorgfältig geprüft, ob und inwieweit Vitamin-D- oder Kalziumsupplemente einzeln und in Kombination zur primären Vorbeugung von Frakturen bei Erwachsenen im Alter über 50 Jahren beitragen, für die kein Risiko für Osteoporose oder Vitamin-D-Mangel besteht [25, 33]. In Anbetracht der begrenzten Daten, die für die Primärprävention verfügbar sind, spricht sich das Gremium gegen eine tägliche Supplementierung mit 400 IE oder weniger Vitamin D und 1000 mg oder weniger Kalzium zur Primärprävention von Frakturen bei Erwachsenen ohne Risiko aus. Für Vitamin-D-Dosen von mehr als 400 IE und für Kalzium-Dosen von mehr als 1000 mg kommt das Gremium zu dem Schluss, dass keine ausreichenden Nachweise vorliegen, um einen Nutzen zu bewerten. Für die höhere Dosis von Vitamin D identifizierte das Gremium beispielsweise eine große Studie mit 4‑monatlichen Gaben von 100.000 IE Vitamin D mit einem signifikanten Nutzen für das Frakturrisiko [34] und eine große Studie mit monatlichen 100.000 IE Vitamin D ohne ein Nutzen für das Frakturrisiko [35].

### Frakturprävention bei Erwachsenen im Alter 50+ mit Vitamin D ohne Kalzium [4]

Die Autoren identifizierten 81 verblindete und nichtverblindete randomisierte Studien bei insgesamt 44.790 Erwachsenen im Alter von 50+. In den berücksichtigten Studien wurden Vitamin D gegen Placebo oder unbehandelte Kontrollen oder Vitamin D in einer anderen Dosierung verglichen. Die Autoren berichten über keinen Effekt der Vitamin-D-Supplementierung auf Frakturen und Stürze. Sie berichten auch über keinen Nutzen von Vitamin D für die Knochenmineraldichte („bone mineral density“, BMD), obgleich bei 3 von 5 Knochendichtemessorten der Nutzen von Vitamin D signifikant war: 0,34 % auf die gesamte Hüftknochendichte (*p* = 0,002), 0,76 % auf den Schenkelhals (*p* < 0,001) und 0,25 % auf die Lendenwirbelsäule (*p* = 0,05), was darauf hindeutet, dass Vitamin D einen, wenn auch kleinen, Nutzen für die Knochendichte hat, und das insbesondere an der Hüfte [4].

Die Bolland-Metaanalyse wurde bezüglich mehrerer Einschränkungen in der Methodik und Interpretation kritisiert und reanalysiert [36]:

- *Erstens *schlossen die Autoren einen wesentlichen Teil der Literatur zu Vitamin D aus, nämlich alle Studien, in denen Vitamin D mit Kalzium kombiniert und mit Placebo verglichen wurde. Solche Studien machen etwa 40 % der qualitativ hochwertigen Daten zur Frakturreduktion aus und haben zu den aktuellen Leitlinien mit einer Empfehlung von 800 IE Vitamin D beigetragen. Das Ausmaß dieser Verzerrung wird in der Metaanalyse 2016 von Weaver et al. beschrieben (s. Abschn. „Metaanalyse Frakturprävention bei Menschen im Alter 65+ bezüglich Vitamin D plus Kalzium“), die eine signifikante Reduktion der gesamten Frakturen um 15 % und eine Reduktion der Hüftfrakturen um 30 % dokumentierte [3].
- *Zweitens* schlagen die Autoren vor, die aktuellen Empfehlungen zu Vitamin D entsprechend ihren Ergebnissen zu überarbeiten. Aktuelle Richtlinien beziehen sich jedoch auf eine tägliche Dosis von 800–1000 IE Vitamin D, während niedrigere Dosen als unwirksam angesehen werden [37, 38]. Außerdem ist gut dokumentiert, dass große jährliche Bolusapplikationen von Vitamin D nicht mehr empfohlen werden, aufgrund eines mehrfach dokumentierten erhöhten Sturz- und Frakturrisikos [32, 39].Die Autoren publizierten eine Reanalyse von 8 randomisierten, placebokontrollierten Studien bezüglich Gesamtfrakturen und 11 placebokontrollierten Studien bezüglich Stürzen, die 800–1000 IE Vitamin D mit mehr als 50 %iger Adhärenz testeten und die großen Bolusstudien ausschlossen. Hier zeigten sich eine signifikante Reduktion der Gesamtfrakturen um 14 % (RR = 0,86, 95 %-KI 0,75–0,98) und eine signifikante Verringerung der Stürze um 12 % (RR = 0,88, 95 %-KI 0,81–0,95; [36]).

Zusammenfassend sind die Limitierungen der Metaanalyse von Bolland et al. wesentlich [36] und es ist zu hinterfragen, inwieweit aus der Bolland-Metaanalyse Konklusionen für die klinische Versorgung ableitbar sind. Tatsächlich lässt sich aus der oben beschriebenen Reanalyse der richtlinienrelevanten Studien ein gegenteiliges Signal ablesen, nämlich dass eine Vitamin-D-Supplementierung in einer täglichen Dosis von 800–1000 IE das Sturz- und Frakturrisiko verringert [36].

### Zusammenfassung zur Frakturprävention

Anhand der 4 neuen Metaanalysen zu Vitamin D und Frakturprävention bei Erwachsenen ist es möglicherweise zu früh, die aktuellen Empfehlungen zu Vitamin D mit oder ohne Kalzium aufzuheben. Insbesondere für ältere Erwachsene mit einem erhöhten Risiko für Frakturen und/oder Vitamin-D-Mangel ist es weiterhin sinnvoll 800–1000 IE Vitamin D/Tag einzunehmen, analog den Empfehlungen der International Osteoporosis Foundation [8], der US Endocrine Society [40] und NOF [41]. Große jährliche Bolusapplikationen von Vitamin D (300.000–500.000 IE) sollten allerdings bei älteren Erwachsenen mit einem Risiko für Frakturen wegen der Zunahme des Frakturrisikos in der klinischen Versorgung nicht fortgesetzt werden. Bei einer größeren monatlichen Dosis von 100.000 IE ist eine weitere Bewertung in Bezug auf Wirksamkeit und Sicherheit erforderlich.

Den aktuellen Empfehlungen zu Vitamin D mit oder ohne Kalzium sollte weiterhin gefolgt werden

Bezüglich Vitamin-D-Supplementation mit oder ohne Kalzium in der Primärprävention von Frakturen, bei Erwachsenen im Alter von 50 und darüber, ohne Vitamin-D-Mangel und ohne Osteoporose, ist anhand der neuen Metaanalysen keine ausreichende Evidenz gegeben. Einschränkend ist jedoch hervorzuheben, dass klinische Studien in dieser Anwendung in der Zahl limitiert sind.

## Was bleibt also vom Hype?

Bezüglich der Knochengesundheit gilt es weiterhin für alle Altersstufen, einen Vitamin-D-Mangel (<20 ng/ml oder <50 nmol/l) zu vermeiden. Die neuen Metaanalysen unterstützen jedoch keinen primärpräventiven Schutz einer Vitamin-D-Supplementation vor Frakturen bei Erwachsenen im Alter 50+ ohne Vitamin-D-Mangel und ohne Osteoporose [24, 25]. Allerdings ist die Zahl von großen Interventionsstudien in dieser Niedrigrisikozielgruppe limitiert [24, 25]. Erwachsenen im Alter von 65 und darüber mit einem hohen Risiko für Vitamin-D-Mangel und Osteoporose sollte eine Vitamin-D-Supplementation mit 800–1000 IE Vitamin D/Tag (mit [26] und ohne [36] zusätzliche Kalziumsupplementation) anhand der bestehenden Evidenz nicht vorenthalten werden. Allerdings sollten bei der Hochrisikopopulation älterer Erwachsener mit erhöhtem Sturzrisiko die großen Vitamin-D-Bolusgaben wegen gegenteiliger Wirkung mit Frakturzunahme vermieden werden [31, 32].

Bezüglich den neuen Resultaten des VITAL mit zusätzlich 2000 IE Vitamin D/Tag zeigten sich eine nichtsignifikante Verringerung der Krebsereignisse und signifikante Verringerung der Krebsmortalität. Bezogen auf die kardiovaskulären Ereignisse fand sich kein Benefit. In der Einordnung der Resultate dieser Studie ist wichtig festzuhalten, dass nur 12 % der Teilnehmer zum Studienbeginn einen Vitamin-D-Mangel hatten und alle Teilnehmer zusätzlich zur Studienmedikation 800 IE Vitamin D einnehmen durften. Damit besteht ein konservativer Bias bezüglich des Wirksamkeitsnachweises von Vitamin D im VITAL. Weitere Forschung, einschließlich ausstehender Evaluation sekundärer Endpunkte von VITAL, ist indiziert, insbesondere bei Menschen mit Vitamin-D-Mangel.

Eine Zusammenfassung der durchgeführten Studien findet sich in Tab. 1.

**Table 1:** 

Autor	Intervention	Zielpopulation und Einschränkungen Methoden	Resultate
**Frakturprävention bei Erwachsenen 50+**			
Weaver et al. (2016, [26]) 8 RCT (*n* = 30.970)	Kombinierter Effekt von Vitamin D plus Kalzium, verglichen zu Placebo	Überwiegend Alter 65+ Jahren Ausschluss von Studien, in denen Vitamin D ohne Kalzium getestet wurde	– 15 %ige Reduktion der Gesamtfrakturen (RR = 0,85, 95 %-KI 0,73–0,98) – 30 %ige Reduktion von Hüftfrakturen (RR = 0,70, 95 %-KI 0,56–0,87)
Zhao et al. (2017, [24]) 33 RCT (*n* = 51.145)	Kalzium und Vitamin D einzeln sowie deren Kombination	Zu Hause lebende Menschen ab 50 Jahren, ohne Osteoporose und ohne Vitamin-D-Mangel – Ausschluss älterer Erwachsener, die in Einrichtungen leben – 11 von 33 mit einer Nachbeobachtungszeit ≤12 Monate 4 Studien ohne Behandlung in der Kontrollgruppe – Keine Anpassung für Adhärenz – 8 der 12 Studien mit Vitamin D in Bolusdosen	Keine signifikante Wirkung von Kalzium oder Vitamin D auf das Risiko einer Hüftfraktur im Vergleich zu Placebo oder keine Behandlung: – Kalzium: RR = 1,53, 95 %-KI 0,97–2,42 – Vitamin D: RR = 1,21, 95 %-KI 0,99–1,47 Kein signifikanter Nutzen bezüglich der Inzidenz von nichtvertebralen, vertebralen oder totalen Frakturen
US Preventive Task Force (2018, [25]) 11 RCT (*n* = 51.419)	Kalzium und Vitamin D einzeln sowie deren Kombination	Zu Hause lebende Menschen ab 50 Jahren, ohne Osteoporose und ohne Vitamin-D-Mangel – Begrenzte Studiendaten für die Primärprävention	Für Vitamin-D-Dosen von mehr als 400 IE (gemäß den aktuellen Empfehlungen) kommen die Autoren zu dem Schluss, dass es nicht genügend Beweise gibt, um einen Nutzen zu bewerten
Bolland et al. (2018) [27] 81 RCT (*n* = 44.790)	Vitamin D im Vergleich zu Placebo, unbehandelten Kontrollen oder einer anderen Dosis Vitamin D	Erwachsene ab 50 Jahren – Die Autoren schlossen Studien aus, in denen Vitamin D mit Kalzium kombiniert wurde, und damit 40 % der Literatur, die zu den aktuellen Richtlinien beitrugen – Die Autoren schlossen große Bolusdosen ein, die das Sturz- und Frakturrisiko konstant erhöht haben – Dosisbewertung, bei der niedrig dosiertes Vitamin D mit 800 IE Vitamin-D-Studien kombiniert wurde	– Die Autoren berichten über keinen Nutzen für die Knochenmineraldichte (BMD), obwohl an 3 von 5 BMD-Stellen der signifikante Benefit BMD: +0,34 % ganze Hüfte (*p* = 0,002), +0,76 % Schenkelhals (*p* < 0,001) und +0,25 % LWS (*p* = 0,05) – Autoren berichten über keinen Nutzen bei Stürzen und Frakturen – Eine Reanalyse [36] der eingeschlossenen Studien mit 800–1000 IE Vitamin D-Studien ohne Bolusstudien zeigte eine signifikante Verringerung der Gesamtfrakturen um 14 % und der Stürze um 12 % hin
**Neue Krebserkrankungen und neue kardiovaskuläre Ereignisse bei Erwachsenen 50+ (VITAL)**			
VITAL [20, 21] untersuchte 25.871 erwachsene Menschen	2000 IE Vitamin D, verglichen zu Placebo, über 5,3 Jahre	12.786 Männer ab 50 Jahren und 13.085 Frauen ab 55 Jahren – Nur 12,7 % der Studienteilnehmer hatten einen Vitamin-D-Mangel mit 25-Hydroxy-Vitamin D-Blutwerten unter 20 ng/ml – Alle Studienteilnehmer konnten zusätzlich 800 IE Vitamin D/Tag einnahmen (auch die Placebogruppe)	*Krebserkrankungen* In der Vitamin-D-Gruppe kleine, nichtsignifikante Verminderung neuer Krebserkrankungen in der gesamten Studienpopulation, signifikante 23 %ige Verminderung neuer Krebserkrankungen bei Subgruppe der afroamerikanischen Studienteilnehmer unter Vitamin-D-Gabe *Krebsmortalität* 17- bis 25 %ige Verminderung der Krebsmortalität in der Vitamin-D-Gruppe *Kardiovaskuläre Erkrankungen*: Kein Benefit von Vitamin D, verglichen zu Placebo

*95* *%-KI* 95 %-Konfidenzintervall, *LWS* Lendenwirbelsäule, *RCT* „randomized controlled trial“, *RR* „risk ratio“

Die Relevanz eines vorbestehenden Vitamin-D-Mangels bezüglich des Wirkungsnachweises einer Vitamin-D-Supplementation bezogen auf Infektionen zeigt sich eindrücklich in einer 2018 publizierten Metaanalyse von 25 randomisierten placebokontrollierten Interventionsstudien zur Vitamin-D-Supplementation bezogen auf das Risiko akuter respiratorischer Infektionen [42]. Dort ergab sich unter Vitamin-D-Supplementation, verglichen zu Placebo, eine Risikoreduktion von akuten respiratorischen Infektionen um 12 % über alle Studienteilnehmer hin weg und um 42 % bei Teilnehmern mit einem Vitamin-D-Mangel beim Studienstart [42].

## Fazit für die Praxis

- Bezüglich der Knochengesundheit gilt es weiterhin für alle Altersstufen, einen Vitamin-D-Mangel zu vermeiden. Erwachsenen ab 65 Jahren mit hohem Risiko für einen Vitamin-D-Mangel und Osteoporose sollte eine Vitamin-D-Supplementation in Form von 800–1000 IE Vitamin D/Tag nicht vorenthalten werden. Bei der Hochrisikopopulation älterer Erwachsener mit erhöhtem Sturzrisiko sind große Vitamin-D-Bolusgaben zu vermeiden, da diese eine gegenteilige Wirkung mit Frakturzunahme zeigen.
- In der Einordnung der Resultate des VITAL ist beachten, dass nur 12 % der Teilnehmer zum Studienbeginn einen Vitamin D‑Mangel aufwiesen und alle Teilnehmer zusätzlich zur Studienmedikation 800 IE Vitamin D/Tag einnehmen durften. Daher ist weitere Forschung, einschließlich der ausstehenden Evaluation der sekundärer Endpunkte von VITAL, indiziert, insbesondere bei Menschen mit Vitamin-D-Mangel.
- Unter der Einnahme von Vitamin D, verglichen mit Placebo, war eine Risikoreduktion von akuten respiratorischen Infektionen um 12 % über alle Studienteilnehmer hinweg und um 42 % bei Teilnehmern mit Vitamin-D-Mangel beim Studienstart zu verzeichnen. Dies verdeutlicht die Relevanz eines vorbestehenden Vitamin-D-Mangels bei der Vorbeugung von Infektionen durch eine Vitamin-D-Supplementation.
